# Mesenchymal stem cells promote glioma neovascularization in vivo by fusing with cancer stem cells

**DOI:** 10.1186/s12885-019-6460-0

**Published:** 2019-12-21

**Authors:** Chao Sun, Xingliang Dai, Dongliang Zhao, Haiyang Wang, Xiaoci Rong, Qiang Huang, Qing Lan

**Affiliations:** 1 Department of Neurosurgery, The Second Affiliated Hospital of Soochow University, 1055, Sanxiang Road, Suzhou, 215004 China; 2 Department of Neurosurgery, the First Affiliated Hospital of Anhui Medical University, Hefei, 230022 China; 3 Department of General Surgery, The Children’s Hospital of Soochow University, Suzhou, 215025 China; 4 Department of Neurosurgery, Dushuhu Public Hospital Affiliated to Soochow University, Suzhou, 215000 China

**Keywords:** Glioma stem cell, Mesenchymal stem cell, Cell fusion, Glioma neovascularization

## Abstract

**Background and objective:**

Tumor angiogenesis is vital for tumor growth. Recent evidence indicated that bone marrow-derived mesenchymal stem cells (BMSCs) can migrate to tumor sites and exert critical effects on tumor growth through direct and/or indirect interactions with tumor cells. However, the effect of BMSCs on tumor neovascularization has not been fully elucidated. This study aimed to investigate whether fusion cells from glioma stem cells and BMSCs participated in angiogenesis.

**Methods:**

SU3-RFP cells were injected into the right caudate nucleus of NC-C57Bl/6 J-GFP nude mice, and the RFP+/GFP+ cells were isolated and named fusion cells. The angiogenic effects of SU3-RFP, BMSCs and fusion cells were compared in vivo and in vitro.

**Results:**

Fusion cells showed elevated levels of CD31, CD34 and VE-Cadherin (markers of VEC) as compared to SU3-RFP and BMSCs. The MVD-CD31 in RFP+/GFP+ cell xenograft tumor was significantly greater as compared to that in SU3-RFP xenograft tumor. In addition, the expression of CD133 and stem cell markers Nanog, Oct4 and Sox2 were increased in fusion cells as compared to the parental cells. Fusion cells exhibited enhanced angiogenic effect as compared to parental glioma cells in vivo and in vitro, which may be related to their stem cell properties.

**Conclusion:**

Fusion cells exhibited enhanced angiogenic effect as compared to parental glioma cells in vivo and in vitro, which may be related to their stem cell properties. Hence, cell fusion may contribute to glioma angiogenesis.

## Background

Glioblastoma (GBM) is the most common and aggressive primary brain tumor in adults. The prognosis of patients remains poor, although its treatment has gradually improved over the years. The progression-free survival of patients with GBM is only six months, with a median survival of 12–15 months.

In addition to radiotherapy and chemotherapy resistance, GBM is characterized by abundant and aberrant vasculature. The microvessel density in glioma tissues increases with the degree of tumor malignancy. The poor prognosis of GBM is closely related to the extent of tumor neovascularization. Hence, the mechanism of glioma angiogenesis and targeted therapy for glioma vasculature are research hotspots.

Jain and Carmeliet [1] described six mechanisms of tumor angiogenesis including classical angiogenesis, vasculogenesis, vasculogenic mimicry (VM), vessel intussusception, tumor cell-endothelial cell co-option, and cancer stem cell-endothelial cell transdifferentiation.

Cancer stem cell-endothelial cell transdifferentiation represents an exciting area of cancer research. Chromosomal disorders of endothelial cells are frequently found in GBM, indicating that cell fusion and re-splitting of fused cells are random and may lead to chromosome loss, recombination and gene reprogramming.

Although cell fusion occurs in various physiological and pathological conditions, its role in cancer biology remains controversial. Cell fusion can occur either between tumor cells, or between tumor cells and normal somatic cells in vivo [2, 3]. Fusion cells are more malignant and display enhanced metastatic ability and drug resistance [4]. In order to verify whether cell fusion is involved in tumor angiogenesis, we co-cultured RFP+ SU3 cells (human glioma cells established in our laboratory) with GFP+ bone marrow mesenchymal cells (BMSCs) reported in our previous studies. The results showed that SU3-RFP and BMSC-GFP can fuse in vitro, and the fused cells can gradually form a vascular structure on Matrigel. Therefore, we hypothesized that glioma stem cells inoculated into nude mice may also fuse with host cells. In the present study, a nude mouse xenograft model using dual-color fluorescent protein tracer was used to isolate fusion cells co-expressing RFP and GFP. Fusion cells from glioma stem cells and BMSCs showed enhanced angiogenesis ability in vivo and in vitro.

## Methods

### Cells and animals

The glioma stem cell line SU3 was established from surgical specimen obtained from a patient with recurrent glioma previously established in our laboratory [5]. Informed consent was obtained from the patient prior to sample acquisition.SU3 cells were regularly authenticated on the basis of morphology, expression of CD133 and nestin, sphere formation ability and tumorigenicity and were regularly examined for (absence of) mycoplasma by Mycoplasma Stain Assay Kit (Beotime, China). SU3 cells were transfected with the RFP gene using a lentiviral-mediated gene transfection kit (GeneChem, Shanghai, China) [6], and cultured in stem cell medium. Briefly, 20 ng/ml bFGF, 20 ng/ml EGF and 1 × B27 supplement were added to DMEM/F12. The NC-C57BL/6 J-GFP nude mice were previously established [7], and raised and bred in the IVC system in SPF environment at the Experimental Animal Center of Soochow University. All animal experiments and operations were approved by the Ethics Committee of the Second Affiliated Hospital of Soochow University.

### Establishment of a dual-color fluorescent protein tracer model

Based on a previous method [7], six-week-old NC-C57BL/6 J-GFP nude mice were anesthetized with pentobarbital sodium (5 mg/100 g) intraperitoneally. Then, 25 μl of SU3-RFP cell suspension (1 × 10^6^) was slowly injected into the right caudate nucleus of the mouse brain using a Hamilton syringe with the assistance of a stereotaxic apparatus. The cranial hole was closed with bone wax, and the scalp was sutured. Fluorescence imaging of transplanted tumors was performed at two and four weeks after inoculation using a small animal imaging system (Kodak, USA). The excitation and emission wavelengths of RFP were 558 nm and 583 nm, respectively, and of GFP were 470 nm and 535 nm, respectively.

### Observation of transplanted tumors and monoclonal RFP+/GFP+ fusion cells

After confirming the transplanted tumor using the small animal imaging system, the mice were sacrificed by cervical dislocaton, and tumor xenografts were washed with phosphate buffered saline (PBS) containing 1% penicillin and streptomycin. The tissues were minced and digested with 0.25% trypsin, and single cell suspension was obtained using brain tumor dissociation kit (Mitsubishi, Germany). After observing RFP+/GFP+ fluorescent cells, the cells were relatively enriched and digested to prepare a single cell suspension, which was subjected to flow cytometry (Beckman, USA). The ratios of RFP+ cells, GFP+ cells and RFP+/GFP+ cells were detected. Cells co-expressing RFP and GFP were separated by flow cytometry. Single RFP+/GFP+ cells with high proliferation ability were monocloned by micropipetting and transferred into 96-well plates for continuous culture. Single cell clones with infinite proliferative capacity were selected and named RFP+/GFP+ fusion cells (FCs). Five mice was used for isolating fusion cells in the present study. The fusion cells isolated from different animals were pooled to study different biological charactersitics and angiogenic ability.

### RT-PCR

The expression of Sox2, Nanog and Oct4 was detected in SU3-RFP, BMSC-GFP and FCs by RT-PCR. The cells in logarithmic growth phase were lysed with Trizol, and total RNA was extracted according to the manufacturer’s protocol. The optical density (OD) value was detected, and the concentration was calculated. Reverse transcription was performed in accordance with the instructions of the reverse transcription kit (Fermentas) to obtain cDNA. 2 μL of cDNA was used for PCR at a total volume of 20 μL. The RT-PCR primers are shown in Table 1.

**Table 1:** Primer sequences and product sizes

Gene	Primer sequence	Product size
Nanog - F	5′-TCTTCCTGGTCCCCACAGTTT-3′	100 bp
Nanog - R	5′-GCAAGAATAGTTCTCGGGATGAA-3′	
Oct-4 - F	5′-CACCATCTGTCGCTTCGAGG-3’	132 bp
Oct-4 - R	5′-AGGGTCTCCGATTTGCATATCT-3’	
Sox2 - F	5′-GCGGAGTGGAAACTTTTGTCC-3’	156 bp
Sox2 - R	5′-GGGAAGCGTGTACTTATCCTTCT-3’	
β-actin - F	5′-CTTTGCAGCTCCTTCGTTG-3’	278 bp
β-actin - R	5′-TGGTAACAATGCCATGTTCA-3’	

### Western blotting

SU3-RFP, BMSC-GFP and FCs in logarithmic growth phase were washed twice with 4 °C pre-cooled PBS. RIPA lysis buffer (Beyotime, Shanghai) was added to fully lyse the cells and obtain total protein using nucleic acid protein analyzer. The protein concentration was determined, and the amount of protein was calculated. The 5× loading buffer and the protein sample were mixed at a ratio of 1:4, and boiled at 100 °C for 5 min to fully denature the protein sample. The protein sample (30 μg/well) was added to a 12% SDS-polyacrylamide gel well for SDS-PAGE at 80 V for 1 h, and the protein was transferred to a polyvinylidene fluoride (PVDF) membrane at 100 V for 1 h. The membrane was blocked with 5% fat-free milk, incubated with RFP (Abcam, 1:1000), GFP (1:2000), CD105 (1:500), Nestin (1:1000), β-actin (1:1000), CD31, CD34 and VE-Cadherin (1:1000) primary antibodies at 4 °C overnight, followed by incubation with horseradish peroxidase-labeled secondary antibody diluted with 5% skimmed milk powder in TBST solution (1:4000 ratio) at room temperature for 30 min, developed with ECL luminescent solution, and analyzed by gel imaging system.

### Immunocytochemical/immunohistochemical staining

Small round slides pre-coated with polylysine were placed on a 24-well plate. 5 × 10^3^ cells in logarithmic growth phase were added to each well, cultured for 12 h, and then fixed with 4% polyoxymethylene. Cells and transplanted tumor paraffin sections were immunolabeled using a previously reported immunocyte/histochemical staining method [8]. After incubation with the primary antibodies (Abcam; Nestin 1:300, CD105 1:200, CD31 1:200, CD34 1:200, VE-Cadherin 1:200, PDGFB 1:100, PDGFR-β 1:100, VEGF 1:100, VEGFR2 1:200, CD90 1:400, CD29 1:100 and CD44 1:200) at 4 °C overnight, the slides were incubated with secondary antibody (Beyotime, Shanghai), stained with diaminobenzidine (DAB), and then counterstained with hematoxylin.

### Tube formation assay in three-dimensional culture

In vitro tube formation assay on Matrigel: Matrigel (BD, USA) and pre-cooled endothelial cell culture medium (DMEM/F12, B27 factor, 10 ng/ml EGF, 5 ng/ml FGF, 10 ng/ml VEGF and 10% fetal calf serum) were added to a 24-well plate and incubated at 37 °C for 30 min. SU3-RFP, BMSC-GFP and FCs in endothelial cell differentiation medium were seeded over solid Matrigel at 5 × 10^4^ cells/well, respectively. After incubation on Matrigel at 37 °C in a 5% CO_2_ chamber, the morphology and tubular structure of the cells were observed under a microscope every 6–8 h.

### Induced endothelial transdifferentiation culture

SU3-RFP, BMSC-GFP and FCs were cultured in endothelial differentiation medium. After 24 h, the cells were centrifuged and collected. The expression levels of relevant endothelial cell markers were measured by Western blotting and immunocytochemical staining. Three groups of cells cultured in DMEM/F12 containing only 10% fetal bovine serum were used as controls.

### Tumorigenicity assay

SU3-RFP and FCs were injected under the right axillary skin of BALB/c athymic nude mice (five mice for each cell type), and the development of subcutaneous tumors was periodically monitored. Tumor tissues were harvested and examined by H&E as well as immunohistochemical staining.

### Detection of microvessel density (MVD) in transplanted tumor

According to the method reported by Weidner et al. [9], the microvessel density (MVD) was tested by immunohistochemical staining of CD31 in transplanted tumors, which was labeled as MVD-CD31. Briefly, the tissue sections were observed under low magnification (100×), areas with the strongest CD31 staining were selected, and numbers of blood vessels in these areas were counted under high magnification (400×). All brown-stained endothelial cells or endothelial cell clusters clearly distinct from adjacent microvessels, tumor cells, and other connective-tissue elements were regarded as one vascular unit, and the average number of blood vessels in 10 visual fields was considered as the vascular density of the tumor. The results were independently judged by two pathologists and then averaged.

### Flow cytometry for detection of stem cell marker expression

SU3-RFP and FCs were digested to form a single cell suspension and adjusted to a concentration of 1–5 × 10^6^ cells/ml using PBS. Cells were resuspended in 200 μl PBS, added into a 1.5 ml EP tube, and incubated in the dark for 30 min at room temperature with antibodies against CD133 (Abcam, Cambridge, UK). Cells were stained with equal amount of isotype control IgG antibody as negative control. After rinsing twice with PBS, flow cytometry was performed on the FACS Canto II (BD Biosciences).

### Statistical analysis

Statistical analysis was performed using SPSS l7.0 software. The results were expressed as mean ± SD. Student’s t-test was used for comparison between the two groups. The data were processed and plotted with GraphPad Prism 5, and *p* < 0.05 indicated statistical significance.

## Results

### Dual-color fluorescent tumor model

**Fig. 1:**
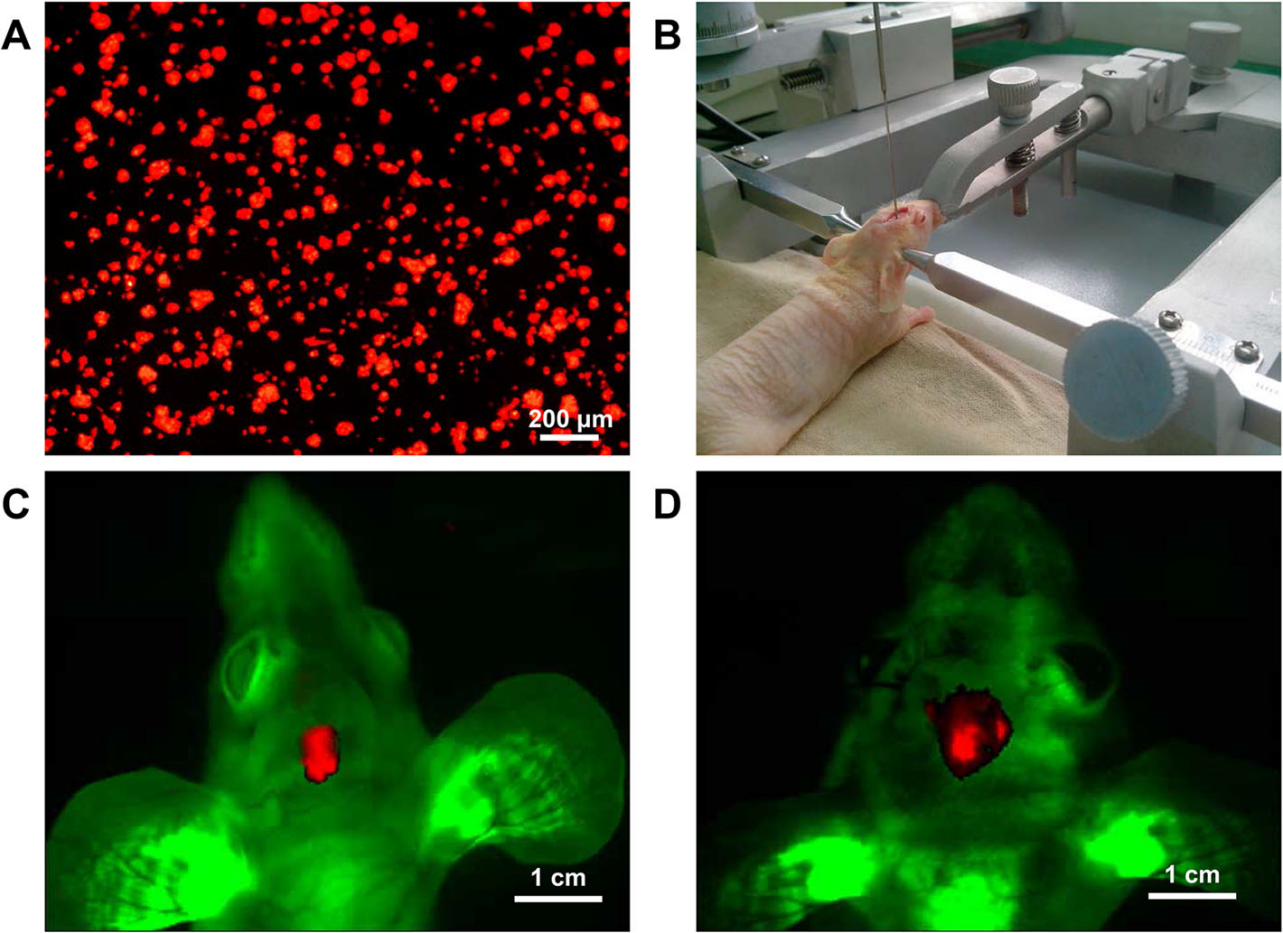
Intracranial transplantation of SU3-RFP cells in GFP nude mice. **a**: SU3 cells were transfected with RFP gene, SU3-RFP cells expressed RFP under fluorescence microscope; **b**: SU3-RFP cell suspension (1 × 10^6^) was injected into the right caudate nucleus of the mice using a Hamilton syringe with the assistance of a stereotaxic apparatus; **c-d**: Whole-body images of tumor-bearing mice on day 14 (**c**) and day 28 (**d**) after transplantation was obtained using the small animal imaging system (Kodak, USA)

SU3-RFP cells (Fig. 1a) were inoculated into GFP nude mouse brain (Fig. 1b). On day 14 and 28, tumors were observed in the mouse brain using the small animal multimodal in vivo imaging system. Against the background of green fluorescent host tissue, a red fluorescent tumor mass was observed at the corresponding site of tumor inoculation (Fig. 1c, d)

### Sorting and cloning of fluorescent cells from transplanted tumors

**Fig. 2:**
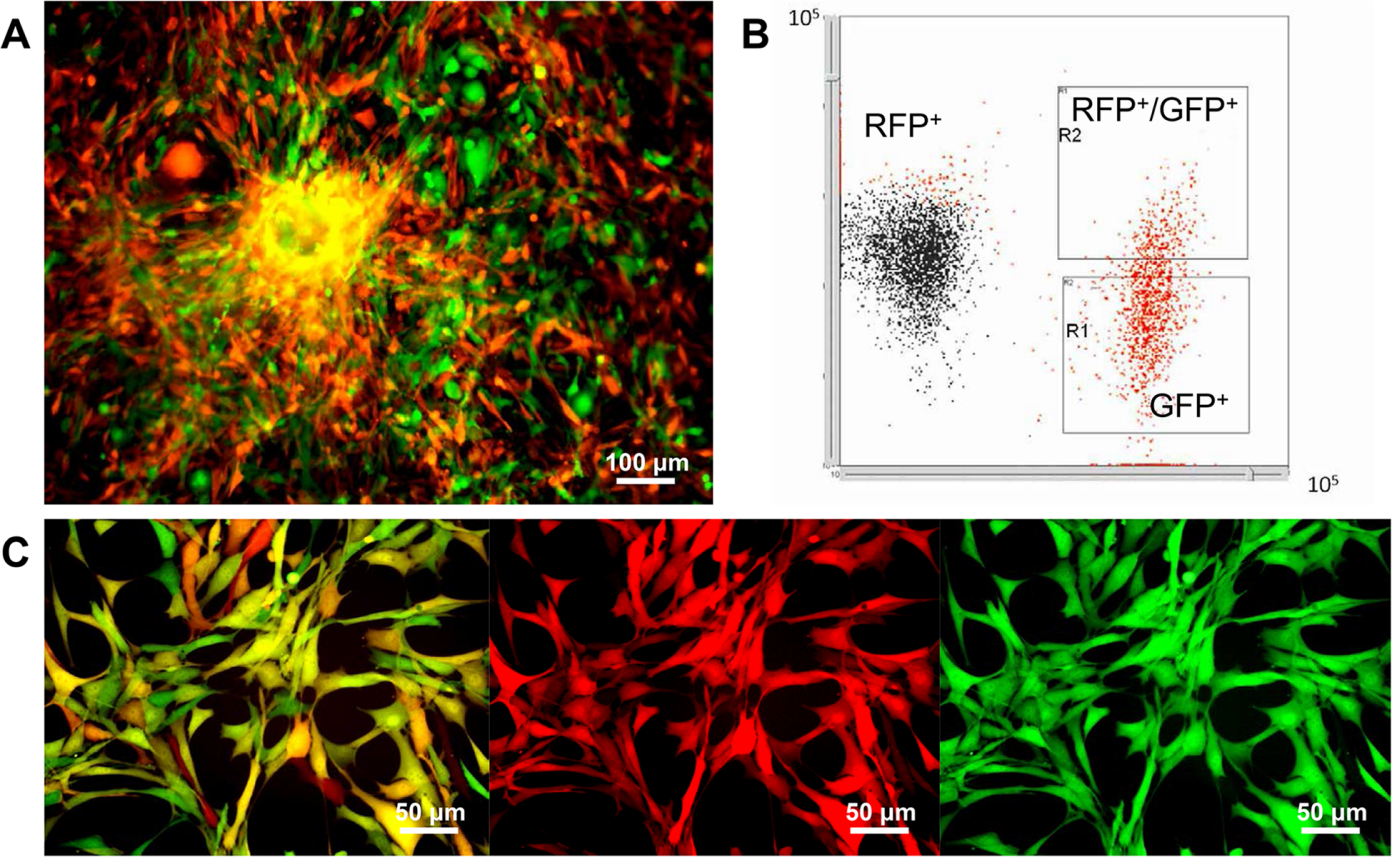
Fluorescent cell sorting. **a**: Fluorescence image of primary culture of SU3-RFP xenograft tumor tissue-derived cells showing both SU3-RFP cells (red) and bone marrow-derived GFP+ cells; **b**: flow cytometry was used to detect the ratio of RFP+, GFP+ and RFP+/GFP+ cells, the ratio of RFP+ cells, GFP+ cells, and RFP+/GFP+ cells was 63.22 ± 5.10%, 28.22 ± 5.96%, and 9.48 ± 1.54%, respectively; **c**: SU3-RFP cells showed red fluorescence, host cells showed green fluorescence. Cells co-expressing RFP and GFP were detected in the merged image indicating tumor-host cell fusion. Scale, A 100 μm; C 50 μm

SU3-RFP cells were transplanted into the brain of GFP nude mice. After 3–4 weeks, the mice were sacrificed by cervical dislocation, and the xenograft tumors were extracted. Primary culture of single cell suspension from the xenograft tumor tissues showed both red and green fluorescent cells (Fig. 2a). The ratios of RFP+ cells, GFP+ cells, and RFP+/GFP+ cells detected by flow cytometry were 63.22 ± 5.10%, 28.22 ± 5.96%, and 9.48 ± 1.54%, respectively (Fig. 2b). The RFP+/GFP+ cells were cloned by our capillary pipette method [7]. Under fluorescence microscope, these cells expressed both RFP and GFP (Fig. 2c).

### Biological characteristics of RFP+/GFP+ cells

**Fig. 3:**
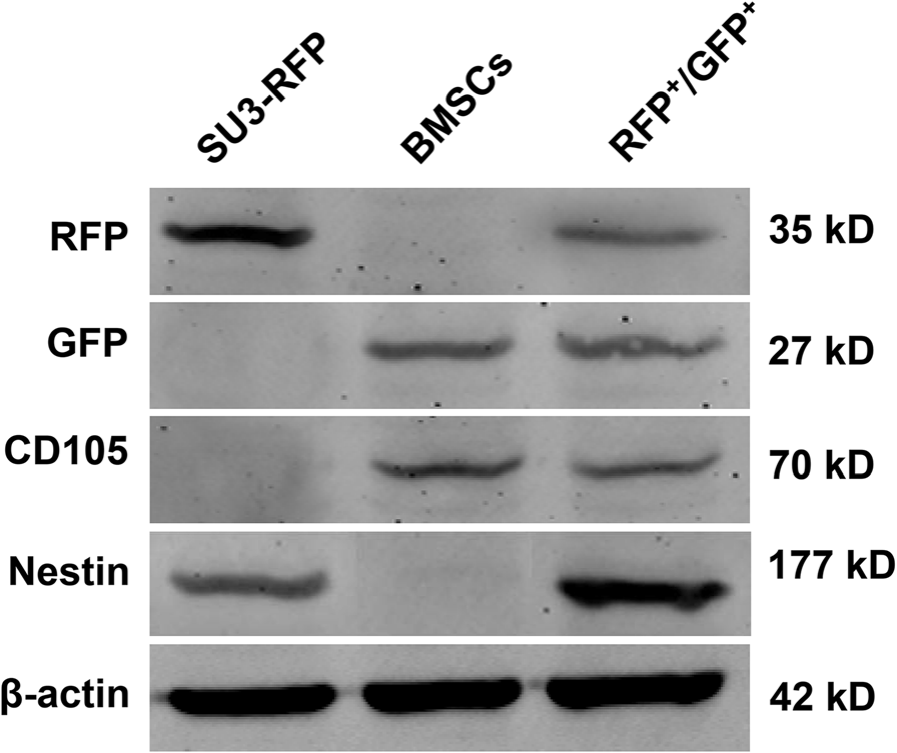
Biological characteristics of the RFP^+^/GFP^+^ cells. Western Blotting was used to detect protein expression levels. The RFP+/GFP+ cells co-expressed RFP and GFP, along with CD105 (marker of BMSCs) and Nestin (marker of SU3-RFP)

RFP+/GFP+ cells co-expressed RFP and GFP by Western Blot, along with CD105 (marker of BMSCs) and Nestin (marker of SU3-RFP) (Fig. 3). Additionally, we performed immunocytochemical staining of BMSC-specific markers, and the results showed that the RFP + GFP+ cells expressed high levels of CD44, CD29 and CD90 (Additional file 1: Figure S1). Therefore, RFP+/GFP+ cells were considered to be fusion of SU3-RFP and BMSCs, and their progeny cells were named fusion cells (FCs).

### Dynamic observation on 3D culture

**Fig. 4:**
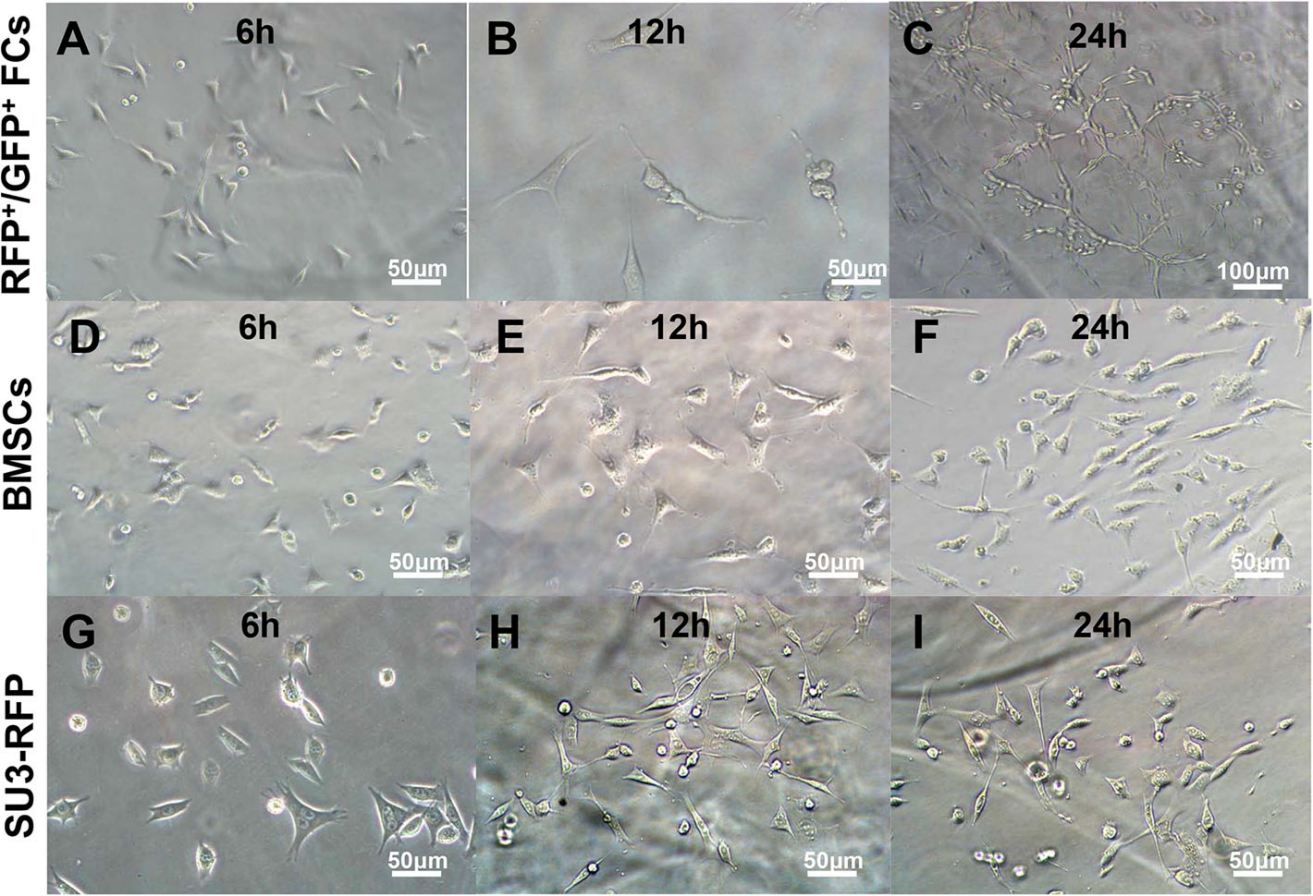
Fusion cells cultured on Matrigel. **a-c**: fusion cells were inoculated on Matrigel, and the cells showed fusiform or star-shaped protrusions at 6 h (**a**). After 12 h, some cell protrusions merged to form a short strip-like structure (**b**). After 24 h, multiple cells were observed to form a circular and tube-like structure (**c**). BMSCs (**d-f**) and SU3-RFP cells (**g-i**) randomly grew in the gel after inoculation, and were disorderly arranged, with no typical lumen structure formation. Scale: **a**-**b**, **d**-**i** 50 μm, **c** 100 μm

The FCs were cultured on Matrigel and underwent continuous morphological changes. They were seeded as single cells, and after six hours, FCs showed fusiform or star-shaped protrusions (Fig. 4a), proliferated further, and some cell protrusions merged to form a short strip-like structure (Fig. 4b). A sparse connection was formed between some cells. After 24 h, multiple cells were observed to form a circular, polygonal and triangular tube-like structure (Fig. 4c). When cultured under standard medium, FCs showed no tubular formation (Additional file 2: Figure S2). BMSCs (Fig. 4d-f) and SU3-RFP cells (Fig. 4g-i) randomly grew in the gel after inoculation, and were disorderly arranged with no typical lumen structure formation.

### Endothelial differentiation culture

**Fig. 5:**
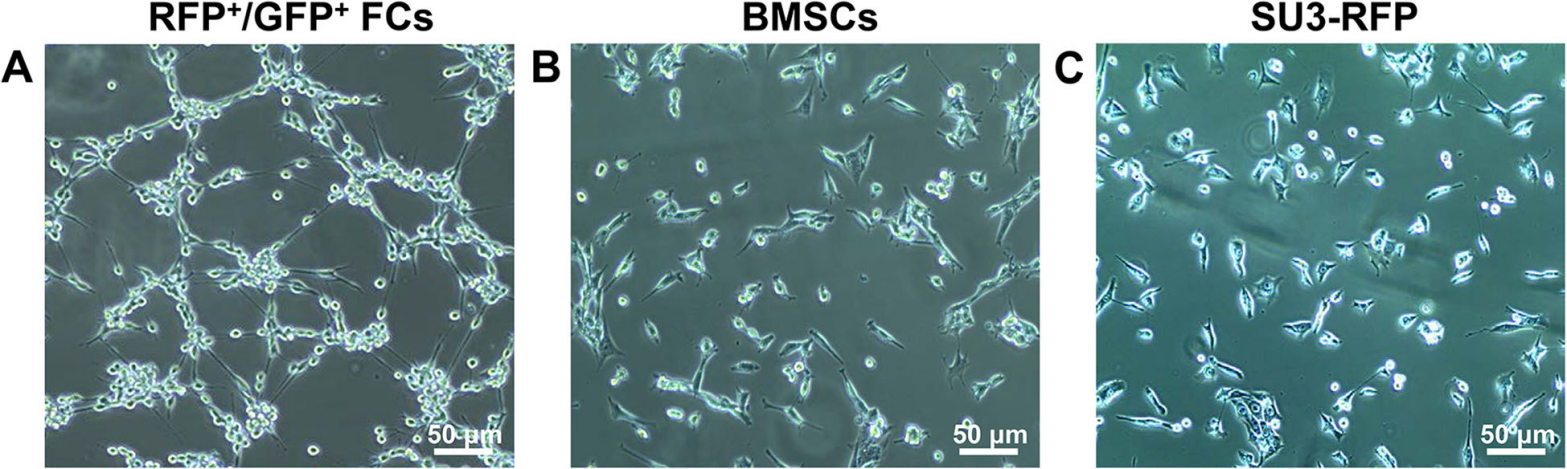
RFP^+^/GFP^+^ FCs, BMSCs and SU3-RFP cells were cultured in endothelial cell differentiation medium for 24 h, the morphology of the three cell types was observed. The proliferation of fusion cells was faster, and the fusion cells showed elongated protrusions, which connected to form a similar network structure (**a**). BMSCs were polygonal and fibrillar (**b**), while SU3-RFP cells showed polymorphism and disorderly growth (**c**). Scale, 50 μm

Cells were cultured on endothelial cell differentiation medium for 24 h. The proliferation of FCs was faster, and they showed elongated protrusions, which connected to form a similar network structure (Fig. 5a). BMSCs and SU3-RFP cells were characterized by their adherent growth. BMSCs were polygonal and fibrillar (Fig. 5b), while SU3-RFP cells differentiated into pleomorphic astrocytoma of its parental phenotype (Fig. 5c), showing polymorphism and disorderly growth. The morphology of cells maintained under standard medium were observed as controls, RFP+/GFP+ FCs grew faster than BMSCs and SU3-RFP, but no lumen-like structure was observed (Additional file 3: Figure S3).

### Vascular endothelial markers expression

**Fig. 6:**
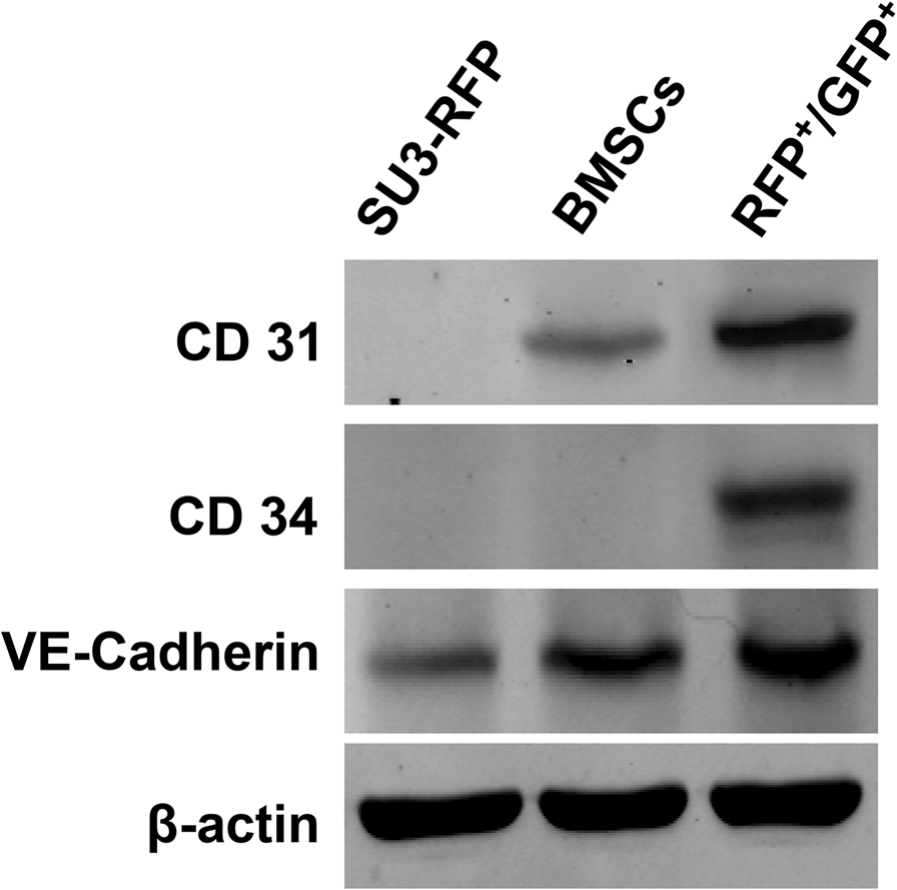
Western Blotting was used to detect protein expression levels. After 24 h of culture in endothelial differentiation medium, fusion cells showed elevated levels of CD31, CD34, and VE-Cadherin (markers of VEC) as compared to SU3-RFP cells and BMSCs

**Fig. 7:**
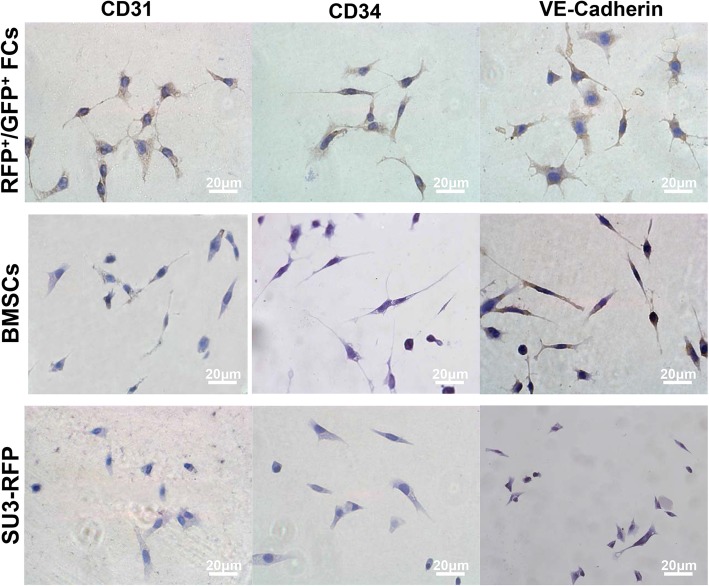
Immunocytochemistry to detect endothelial markers in fusion cells, BMSCs and SU3-RFP cells. Endothelial cell markers were absent in SU3-RFP cells after 24 h of stimulation in endothelial cell culture environment. BMSCs partially expressed VE-Cadherin and CD31, while majority of the FCs expressed CD31, CD34 and VE-Cadherin. Scale, 50 μm

The expression of CD31, CD34 and VE-Cadherin was significantly increased in FCs after endothelial differentiation. Weak expression of VE-Cadherin and CD31 was detected in BMSCs, while weak expression of VE-Cadherin was detected in SU3-RFP cells (Fig. 6). Similarly, immunocytochemical results showed no expression of endothelial cell markers in SU3-RFP cells after 24 h of stimulation in endothelial cell culture environment. BMSCs partially expressed VE-Cadherin and CD31, while majority of the FCs expressed CD31, CD34 and VE-Cadherin (Fig. 7), confirming that the endothelial cell culture environment can significantly promote the differentiation of FCs into endothelial cells (Fig. 7). FCs, BMSCs and SU3-RFP cells cultured in normal medium did not express CD31, CD34 or VE-Cadherin in the control group (Additional file 4: Figure S4). Since VEGF and PDGF-B play an important role in glioma angiogenesis. Hence, we evaluated the expression of PDGF-B and VEGF together with their receptors in FCs by immunocytochemical staining. Strong cytoplasmic expression of PDGF-B/PDGFR-β and VEGF/VEGFR2 were detected in FCs (Additional file 5: Figure S5).

### In vivo tumorigenicity assay

**Fig. 8:**
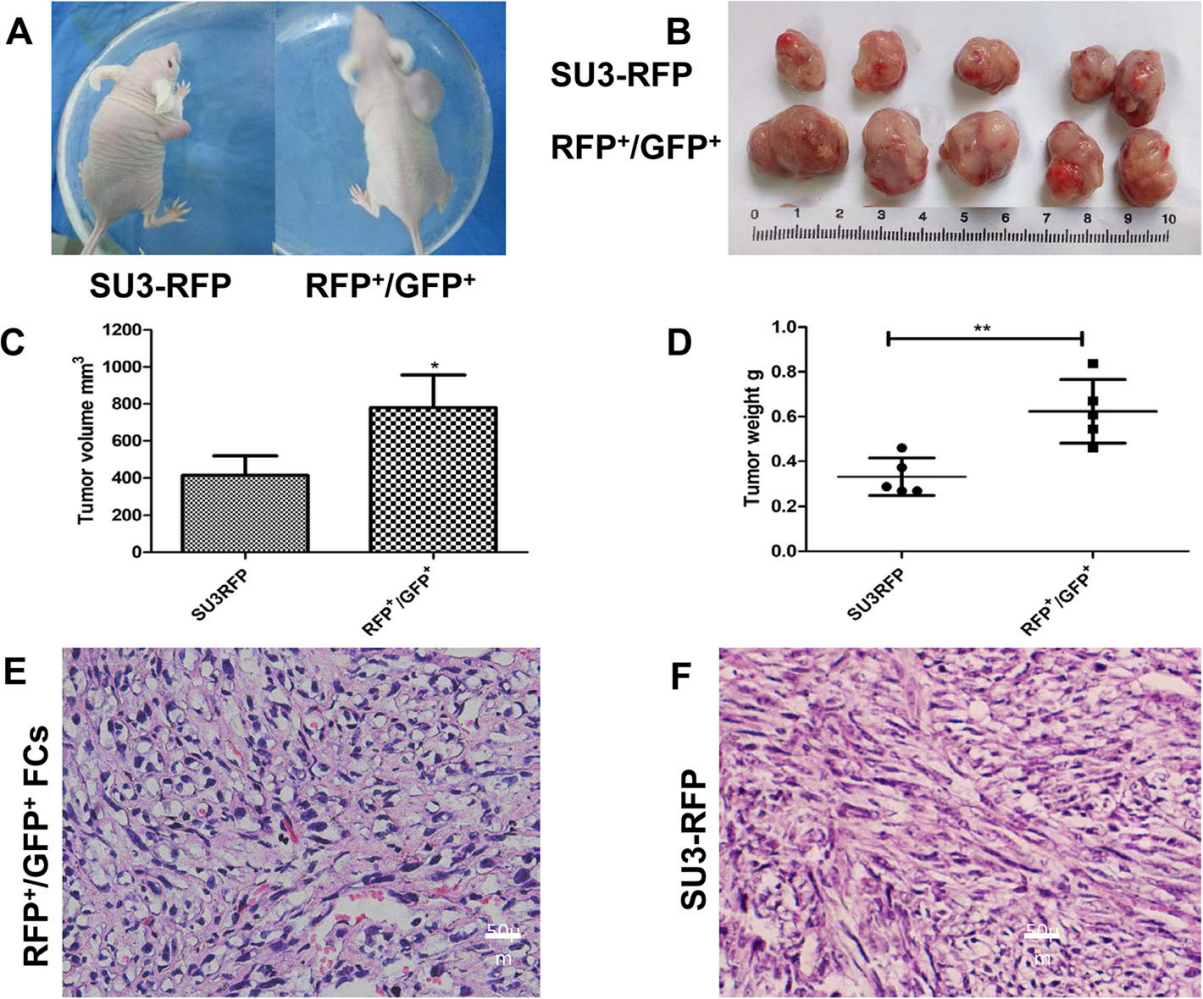
In vivo tumorigenicity. **a**: Representative image of a nude mouse transplanted with SU3-RFP cells and fusion cells; **b**: Representative image of subcutaneous xenografts; **c**: Transplant tumor weight statistics, the average volume of the transplanted tumor was 779.6 ± 177.78 mm^3^ in the FC group and 414.01 ± 105.04 mm^3^ in the SU3-RFP group. **d**: Transplant tumor volume statistics, the average weight of the tumor was 0.62 ± 0.14 g in the FC group and 0.33 ± 0.08 g in the SU3-RFP group. **e**: HE staining of fusion cell xenograft, more proliferative vascular structures were detected as compared to SU3-RFP transplant tumor (**f**). Scale, E and F, 20 μm

The tumorigenic rate of FCs and SU3-RFP cells in nude mice was 100% (5/5). On day 30 after subcutaneous inoculation, the mice were sacrificed by cervical dislocation, and the subcutaneous xenografts were isolated, measured and weighed. The volume and weight of the FC group were larger than that of the SU3-RFP group. The average volume of the transplanted tumor was 779.6 ± 177.78 mm^3^ in the FC group and 414.01 ± 105.04 mm^3^ in the SU3-RFP group. The average weight of the tumor was 0.62 ± 0.14 g in the FC group and 0.33 ± 0.08 g in the SU3-RFP group (Fig. 8a-d). HE staining of SU3-RFP subcutaneous xenografts showed that the cells were closely arranged, with large nuclei, irregular and deep staining. In the xenografts of FCs, tumor cells with high atypia, increased nuclear-to-cytoplasmic ratio and more proliferative vascular structures were detected (Fig. 8e, f).

### Microvessel density of intracranial xenografts

**Fig. 9:**
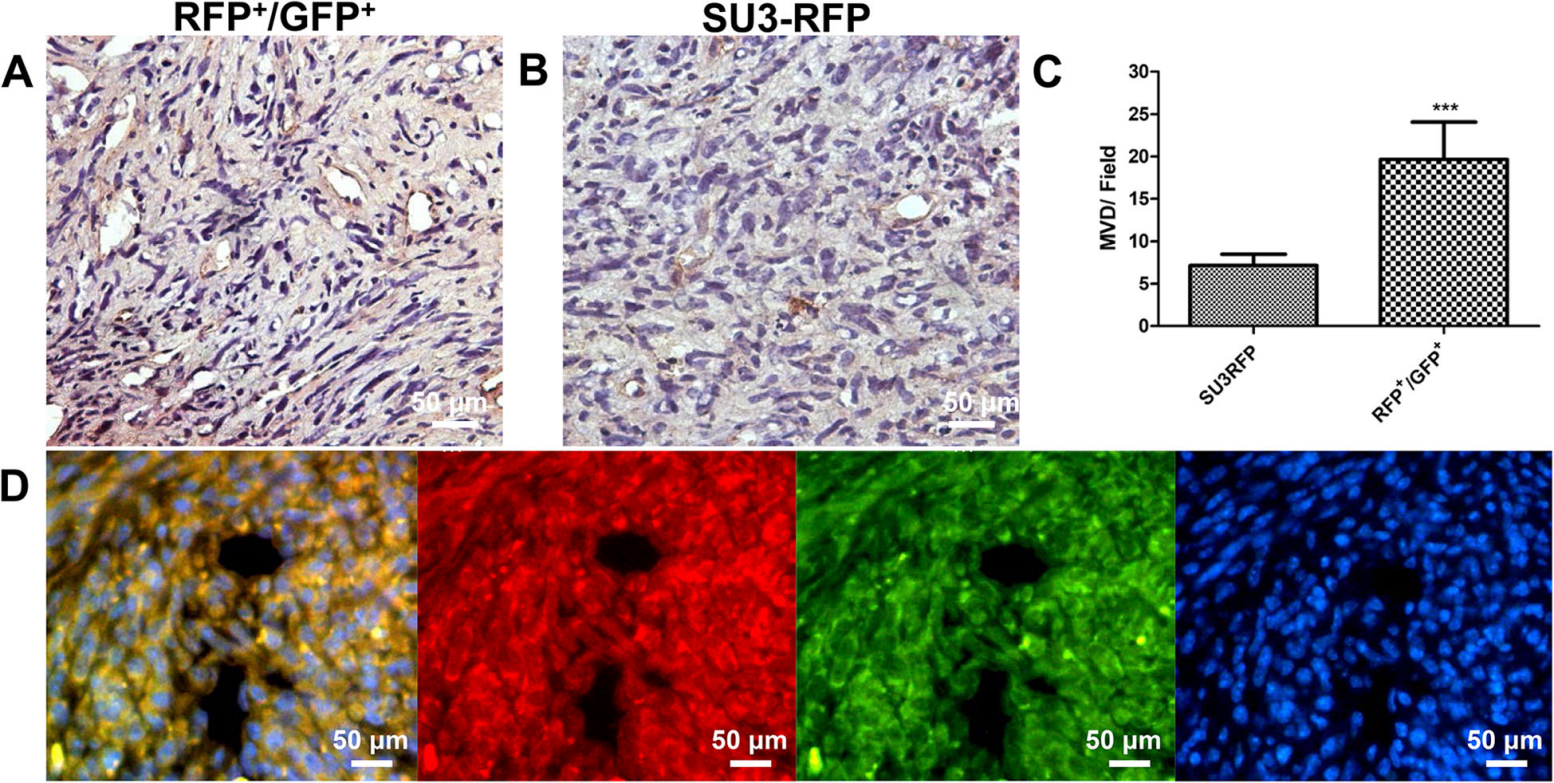
Immunohistochemistry to detect CD31 expression in fusion cell xenograft (**a**) and SU3-RFP xenograft (**b**); **c**: Microvessel density comparison between the two groups, MVD-CD31 was 19.67 ± 1.8 vessels in RFP+/GFP + cells and 7.16 ± 0.54 vessels in SU3-RFP cells, respectively; **d**: Fusion cell xenografts observed under fluorescence microscopy. Scale, A and B 20 μm, D 50 μm. ***p < 0.001

RFP+/GFP+ cells and SU3-RFP cells were injected into the brain of nude mice, respectively. Microvessel densities of the xenograft tumors were tested by immunohistochemistry (IHC) staining of CD31. The MVD-CD31 was 19.67 ± 1.8 vessels in RFP+/GFP+ cells and 7.16 ± 0.54 vessels in SU3-RFP cells, respectively. MVD was significantly greater in the RFP+/GFP+ cells xenograft tumor as compared to that in the SU3-RFP xenograft tumor (*p* < 0.001) (Fig. 9a-c). Frozen sections of the FC-transplanted tumor showed a vascular cavity-like structure under fluorescence microscope (Fig. 9d).

### Stem cell features of fusion cells

**Fig. 10:**
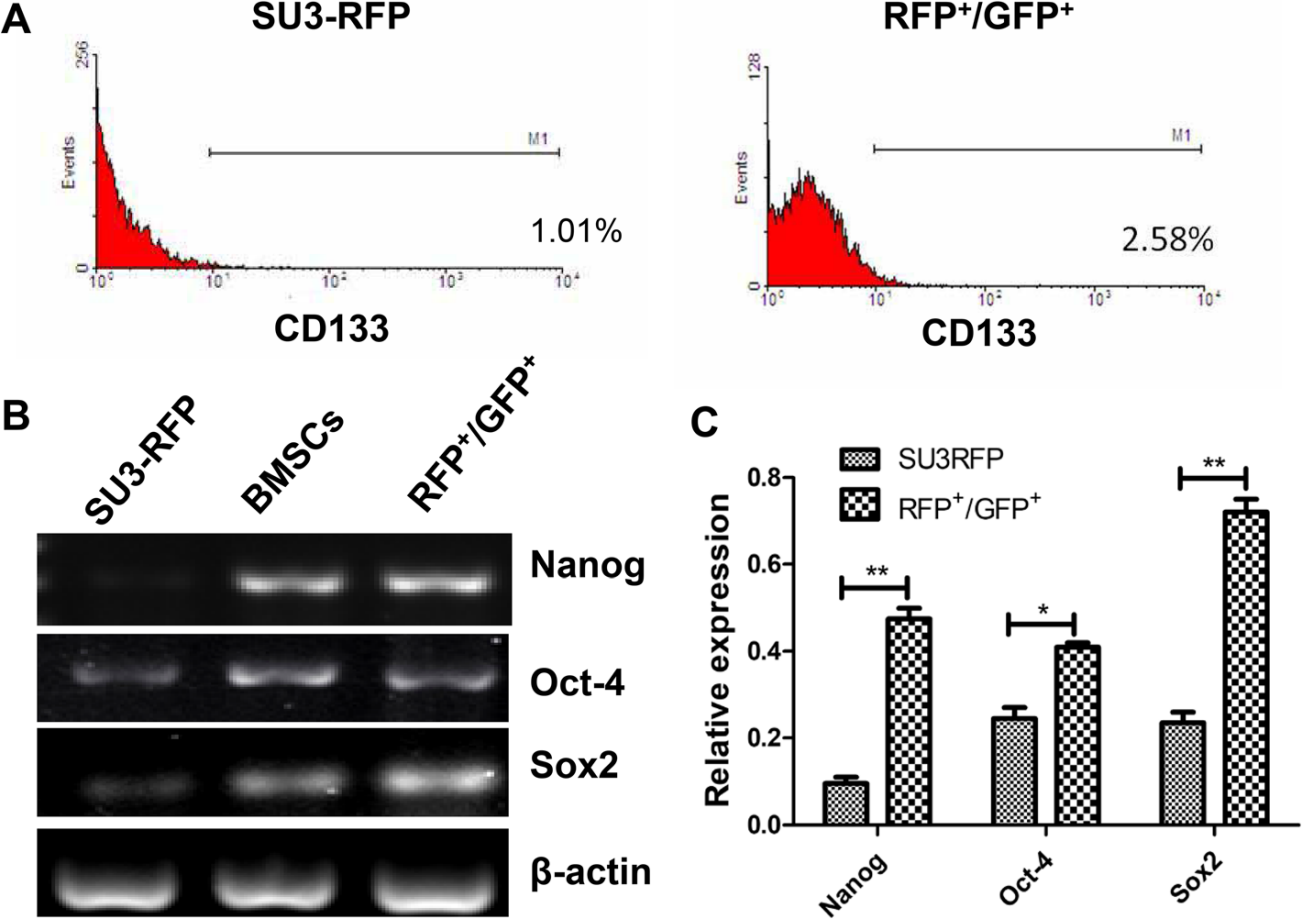
Stem cell features of fusion cells. **a**: the ratio of CD133+ cells in FCs was 2.67 ± 0.35%, and the ratio of CD133+ cells in SU3-RFP was 1.05 ± 0.18% by flow cytometry; **b-c**: RT-PCR showed that the expression of stem cell-associated transcription factors Oct4, Sox2, and Nanog was also increased

CD133 is considered to be a marker of cancer stem cells. The expression of CD133 on FCs and glioma cells was assessed by flow cytometry. The results showed that the ratio of CD133+ cells in FCs was 2.67 ± 0.35%, and the ratio of CD133+ cells in SU3-RFP was 1.05 ± 0.18% (Fig. 10a). The expression of CD133 on FCs was higher. The expression of stem cell-associated transcription factors Oct4, Sox2 and Nanog was also increased in FCs (Fig. 10b, c).

## Discussion

The cellular and molecular mechanisms of glioma angiogenesis are research hotspots in glioma biology. Various mechanisms of glioma angiogenesis have been elucidated, but there remains considerable controversy [10].

According to the classical hypothesis proposed by Folkman, cells responsible for glioma neovascularization should be derived from vascular endothelial cells that are induced by glioma cells [11], a process known as angiogenesis. Another source of glioma neovascularization is the formation of neovascularization by bone marrow-derived endothelial progenitor cells recruited by tumor cells [12], a process known as vasculogenesis.

The mechanism by which tumor stem/progenitor cells participate in tumor angiogenesis has been extensively studied. Dong [13], Lucia Ricci-Vitiani [14], and Wang R [15] reported that glioma stem cells can transdifferentiate into endothelial cells and directly participate in the formation of tumor blood vessels, but these findings were contradicted by other reports. Cheng et al. found no glioma stem cell-derived endothelial cells in glioma xenografts. Instead, they found that majority of the pericytes (57–89%) are derived from glioma stem cells [16]. GaoLiang Ouyang suggested that it may be due to the fusion of glioma stem cells and endothelial cells or pericytes [17].

Although cell fusion is a normal physiological process that occurs in diverse organisms and plays essential roles in fertilization and development of various organ systems, its role in carcinogenesis and tumor progression remains controversial. There is considerable evidence to suggest that cell fusion can occur either between tumor cells in vivo or between tumor cells and normal somatic cells [2, 3]. The target cells for tumor cell fusion remain unknown. BMSCs have been studied in the context of cell fusion. They possess properties of mesenchymal cells and stem cells. Moreover, BMSCs have been confirmed to participate in tumor microenvironment, and can be recruited by tumor cells. Tumor cells can acquire high expression of molecules and proteins related to stem cell-like properties by fusing with mesenchymal stem cells. BMSCs can spontaneously fuse with breast cancer, prostate cancer, ovarian cancer, lung cancer and other cells in vivo and in vitro [18–20], but have been rarely reported to fuse with glioma cells.

Our previous study found that glioma stem cell SU3-RFP and BMSCs can be fused in vitro [8], and the fused cells display angiogenic properties. Whether glioma cells and mesenchymal stem cells can fuse in vivo remains unknown, and the properties of these cells after cell fusion need to be examined. In the present study, we isolated fusion cells expressing RFP and GFP in the dual-color orthotopic xenograft glioma specimens, and found that these cells exhibited enhanced angiogenic effect in vivo and in vitro, and promoted tumor growth.

BMSCs are one of the most critical components of tumor microenvironment, and have been recently detected in brain tissue [21], glioma mouse orthotopic transplantation tumor model [22], and human glioma clinical specimens [23, 24]. These studies confirmed that BMSCs participate in the microenvironment of glioma, but their functional contributions to tumor angiogenesis and growth are poorly understood.

The role of BMSCs in tumor growth and angiogenesis is very complicated. BMSCs have been described as a double-edged sword for tumors [25]. BMSCs can synthesize and secrete various cytokines to act on endothelial cells, such as VEGF, PDGF, bFGF, angiopoietin, IL-6, IL-8 and TGF-β, which promote tumor angiogenesis [26]. Suzuki K found that subcutaneous co-injection of tumor cells and BMSCs in mice resulted in more rapid tumor growth and greater tumor vessel area as compared to injection of tumor cells alone in assays using either B16-LacZ or LLC cells [27]. However, some scholars believed that BMSCs can inhibit tumor angiogenesis [28, 29]. In a study of glioma models, Ho et al. found that subcutaneous co-inoculation of BMSCs and glioma cells in nude mice significantly inhibited tumor growth and microvessel density as compared to inoculation of glioma cells alone [30]. Co-culture of BMSCs and glioma cells was found to inhibit angiogenesis in gliomas by down-regulating the expression of angiogenic markers such as PDGF-BB, IGF-1, FGF-2, and IL-1b [30]. Recent studies indicated that the role of BMSCs in angiogenesis mainly depends on paracrine effects on other cells. However, some studies found that BMSCs directly support the tumor vasculature by differentiating into endothelial cells, pericytes or other types of cells [31, 32]. Whether BMSCs can directly transform into endothelial cells in the glioma microenvironment remains unknown. However, the present study found that BMSCs can fuse with glioma cells in vivo and transdifferentiate into endothelial-like cells. Additionally, fusion cells showed enhanced proliferation and tumorigenic potential. The wound scratch assays revealed that fusion cells own increased migration ability (Additional file 6: Figure S6).

Cell fusion facilitates tumor progression [4]. In 1985, LaGarde and Kerbel [33] proposed that cell fusion may lead to important changes in tumor cell gene expression, which is now called gene reprogramming. Theoretically, the parent-derived genome can recombine in the fusion cells, wherein the parental glioma cells confer tumorigenicity to the fusion cells, while the BMSCs confer stem cell characteristics. The differentiation ability of adult stem cells suggested reprogramming of mature cells after fusion of stem cells and differentiated mature cells. Stem cells acquire other phenotypes through cell fusion. Similarly, the plasticity of cancer stem cells may also be cell fusion-dependent.

The occurrence and development of tumors are closely linked to tumor angiogenesis, especially when the tumor’s own blood supply cannot meet the rapid growth of tumor cells. Tumor cells may fuse with stem cells and regain self-renewal through dedifferentiation or gene reprogramming, and subsequently differentiate into endothelial cells or pericytes to form new blood vessels.

CD133 is believed to be a valuable marker for cancer stem cells in solid tumors including GBM [34]. We found that the ratio of CD133+ cells was significantly higher among fusion cells than among the parental glioma cells by flow cytometry. Nanog, Oct-4 and Sox2 are important stem cell transcription factors for stem cell regulation and maintenance. Previous studies had confirmed that BMSCs express high levels of Nanog, Oct-4, and Sox2 [35], which was consistent with our results. We found that the expression of Nanog, Oct-4 and Sox2 was higher in fusion cells as compared to the parental glioma cells by RT-PCR. Therefore, BMSCs may assign the genes encoding the above transcription factors to fusion cells through cell fusion. Fusion cells have enhanced stem cell characteristics, are more plastic, can undergo phenotypic transformation to differentiate into endothelial cells, and participate in tumor microcirculation.

## Conclusions

Our previous study demonstrated the dynamic process of cell fusion between human glioma cells and BMSCs in a co-culture system. In the present study, fusion cells were isolated from dual-color orthotopic model of transplantable xenograft glioma, and showed enhanced angiogenic ability as compared to the parental tumor cells in vitro and in vivo. Moreover, cell fusion could enhance tumor angiogenesis, and a new target for anti-tumor angiogenesis therapy was proposed. Future studies should dynamically trace when and how tumor cells fuse with host cells in vivo, as well as the dynamic evolution of cell fusion and how it participates in the process of vascular architecture.

## Supplementary information

**Additional file 1: Figure S1.** Representative image of immunocytochemistry staining of BMSC cell surface markers in fusion cells. As shown, fusion cells expressed high levels of CD90, CD44 and CD29 (Scale: 50 μm).

**Additional file 2: Figure S2.** In the 3D culture, RFP+/GFP+ FCs maintained under standard medium showed no tubular formation.

**Additional file 3: Figure S3.** The morphology of cells maintained under standard medium. RFP+/GFP+ FCs grew faster than BMSCs and SU3-RFP, but no lumen-like structure was observed.

**Additional file 4: Figure S4.** FCs, BMSCs and SU3-RFP cells cultured in normal medium did not express CD31, CD34 or VE-Cadherin in the control group.(Scale: 20 μm)

**Additional file 5: Figure S5.** We evaluated the expression of PDGF and VEGF together with their receptors in fusion cells, strong cytoplasmic expression of PDGF-B/PDGFR-β and VEGF/VEGFR2 was detected in fusion cells.

**Additional file 6: Figure S6.** Cell monolayers were wounded by scraping with a sterile pipette tip, then washed twice to remove detached cells and debris, and the size of wound was observed every 6 h. The results revealed that fusion cells possessed increased migration ability.

## Data Availability

The datasets used and/or analyzed in the current study are available from the corresponding author on reasonable request.

## Abbreviations

BMSCs: Bone marrow mesenchymal cells
DAB: Diaminobenzidine
DAPI: 4,6-diamidino-2-phenylindole dihydrochloride
FCs: Fusion cells
GBM: Glioblastoma
IHC: Immunohistochemistry
MVD: Microvessel density
OD: Optical density
PBS: Phosphate buffer
PVDF: Polyvinylidene fluoride
VM: Vasculogenic mimicry
