# Supplementary figures and images for: Mesenchymal stem cells promote glioma neovascularization in vivo by fusing with cancer stem cells
Source: BMC Cancer. 2019 Dec 21;19:1240. doi: 10.1186/s12885-019-6460-0 (PMC6925905; doi:10.1186/s12885-019-6460-0)

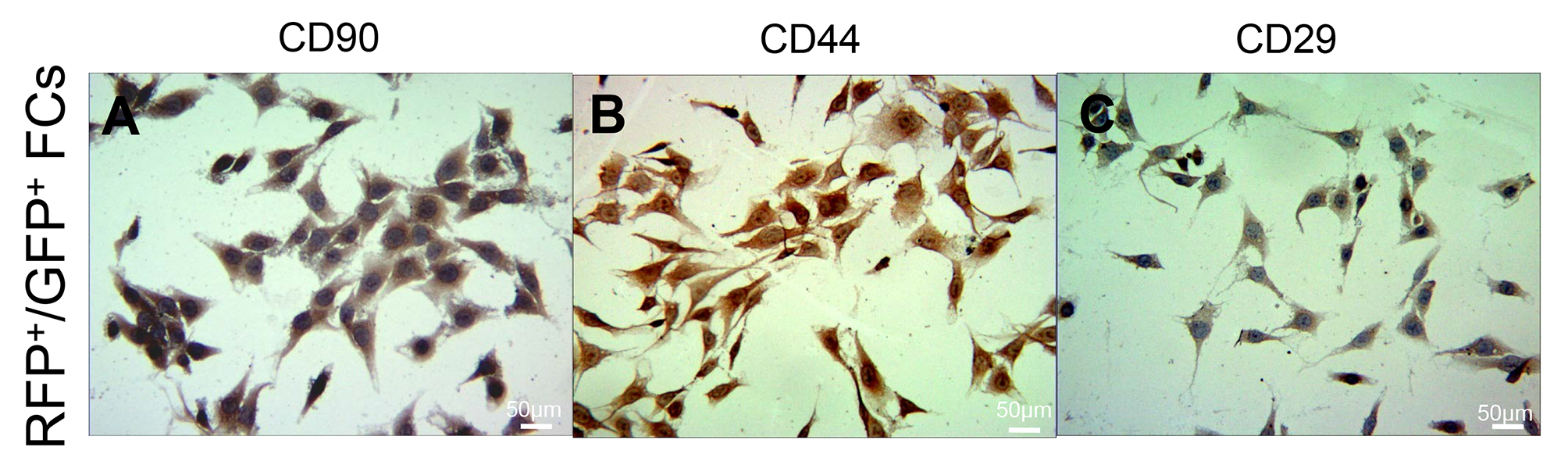

Supplement: Supplementary file 1 — Additional file 1: Figure S1. Representative image of immunocytochemistry staining of BMSC cell surface markers in fusion cells. As shown, fusion cells expressed high levels of CD90, CD44 and CD29 (Scale: 50 μm). [file 12885_2019_6460_MOESM1_ESM.tif]

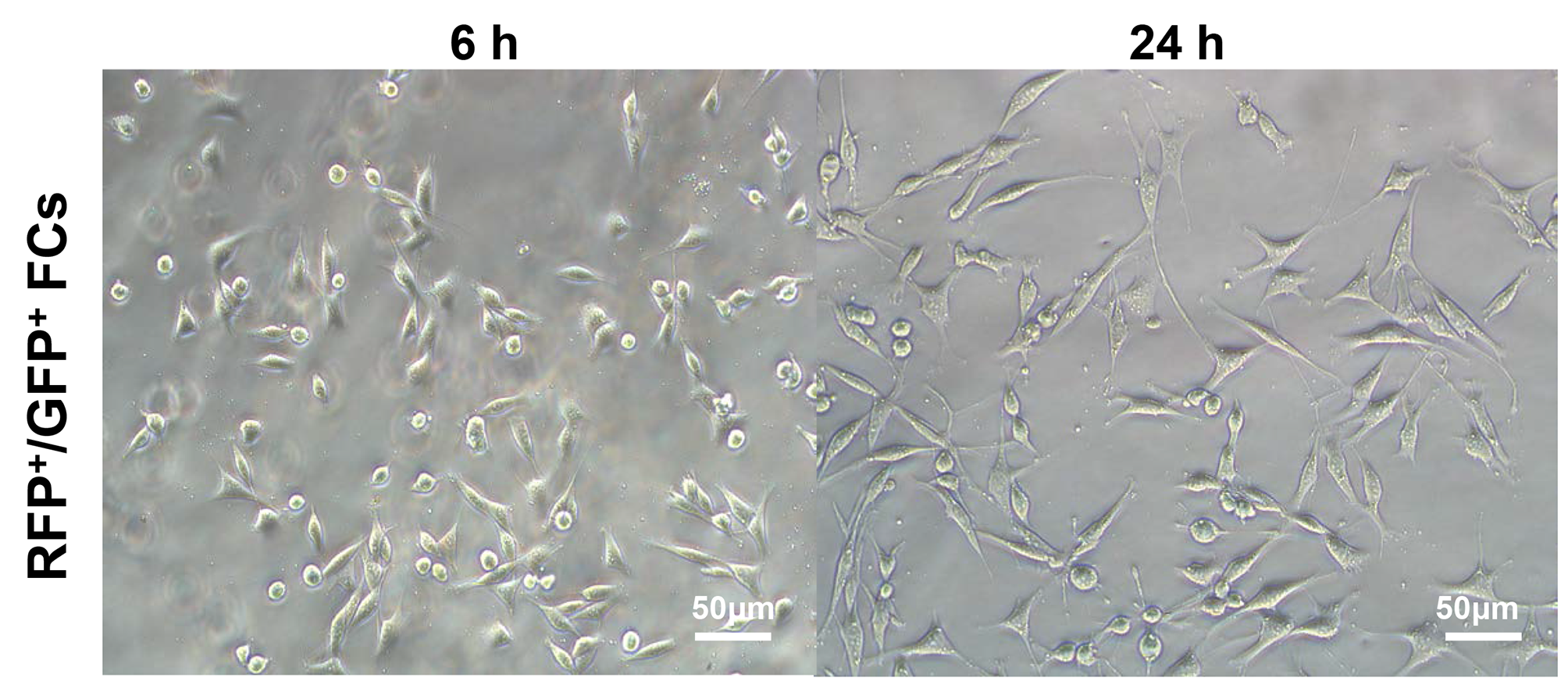

Supplement: Supplementary file 2 — Additional file 2: Figure S2. In the 3D culture, RFP+/GFP+ FCs maintained under standard medium showed no tubular formation. [file 12885_2019_6460_MOESM2_ESM.tif]

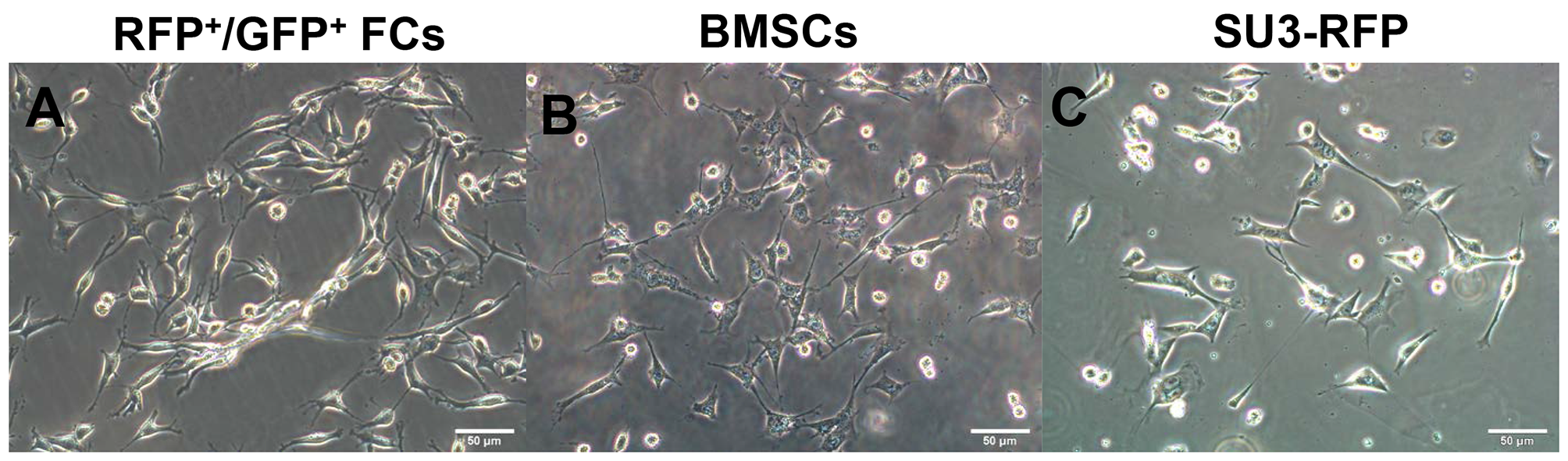

Supplement: Supplementary file 3 — Additional file 3: Figure S3. The morphology of cells maintained under standard medium. RFP+/GFP+ FCs grew faster than BMSCs and SU3-RFP, but no lumen-like structure was observed. [file 12885_2019_6460_MOESM3_ESM.tif]

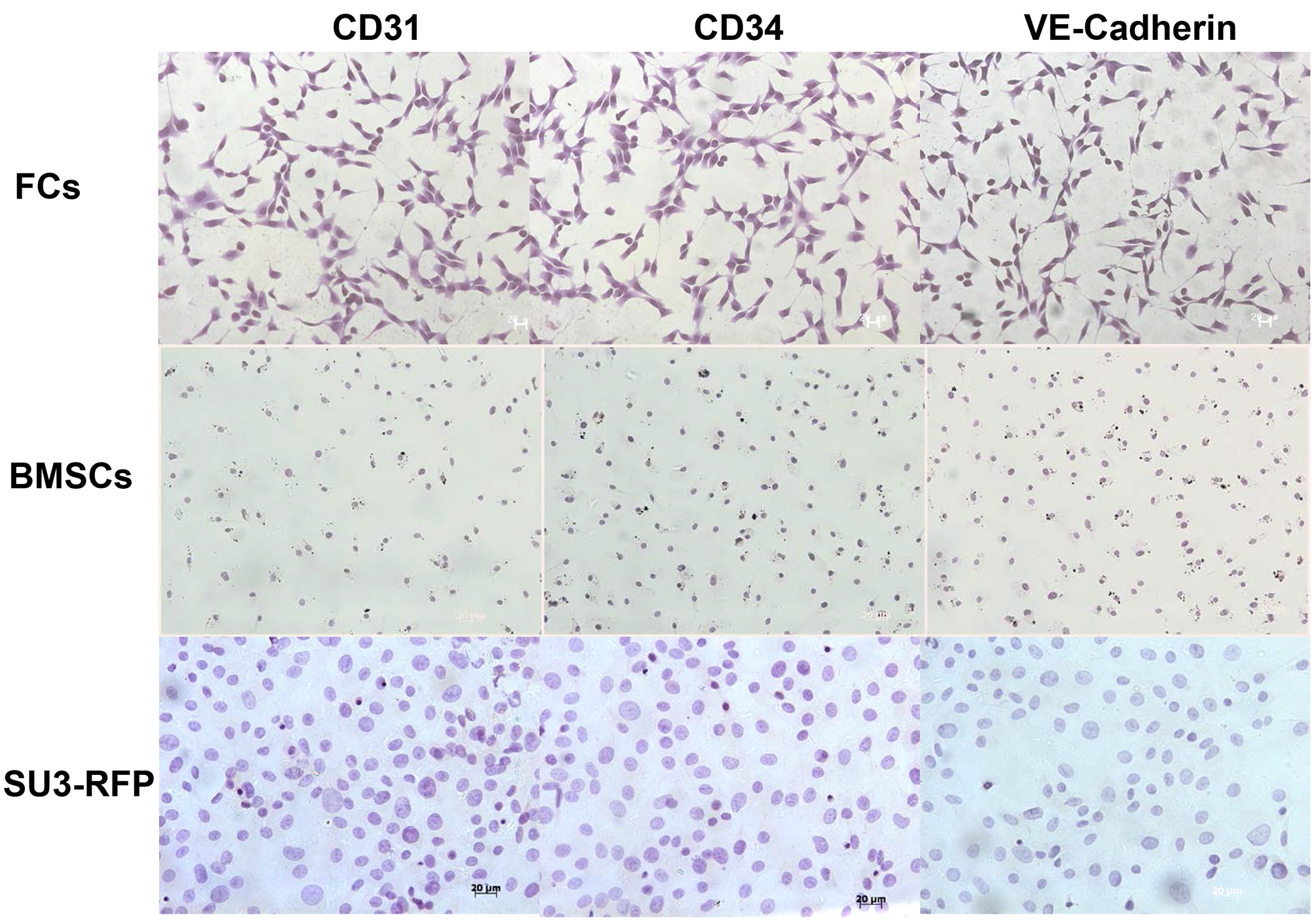

Supplement: Supplementary file 4 — Additional file 4: Figure S4. FCs, BMSCs and SU3-RFP cells cultured in normal medium did not express CD31, CD34 or VE-Cadherin in the control group.(Scale: 20 μm) [file 12885_2019_6460_MOESM4_ESM.tif]

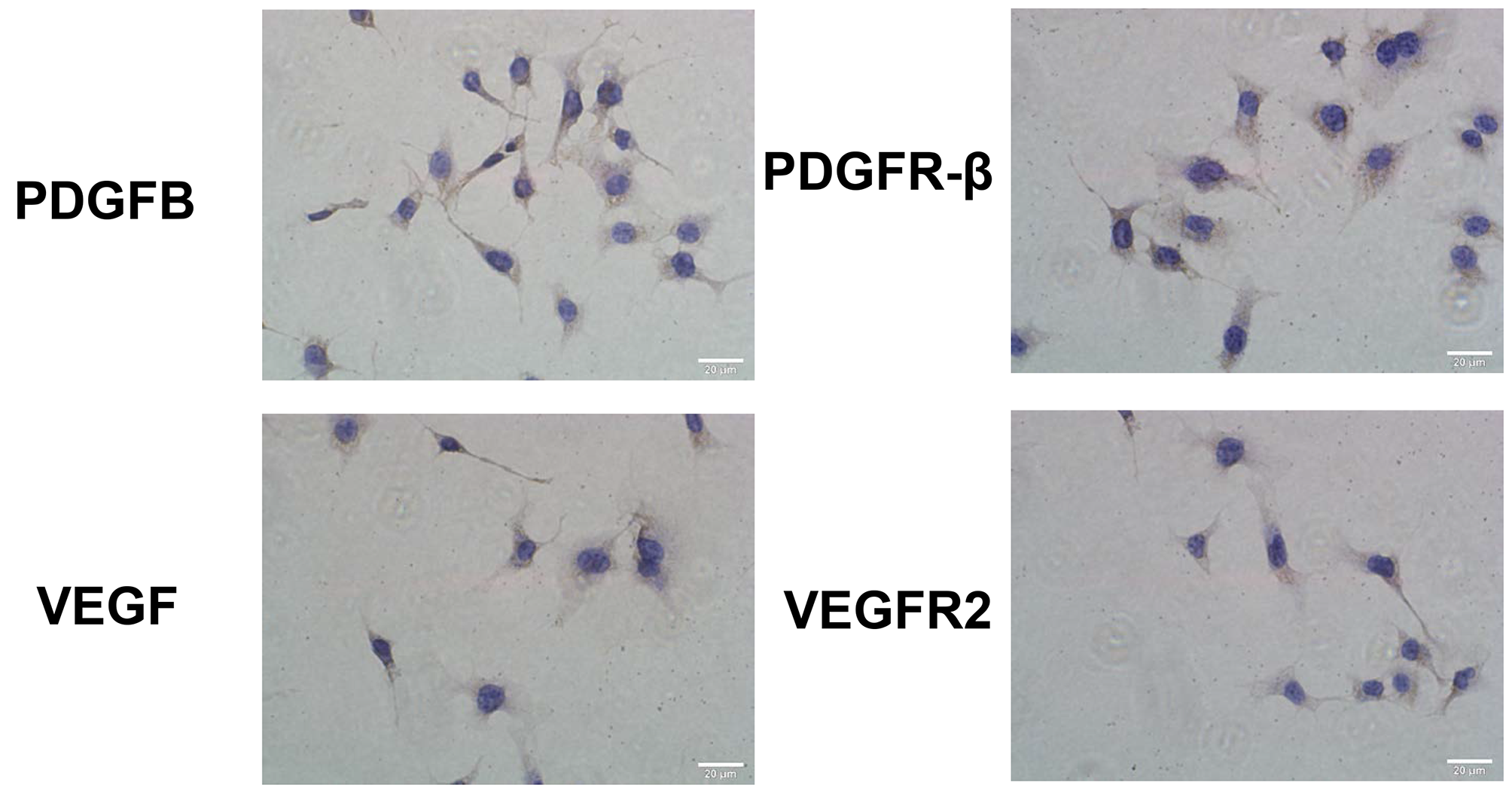

Supplement: Supplementary file 5 — Additional file 5: Figure S5. We evaluated the expression of PDGF and VEGF together with their receptors in fusion cells, strong cytoplasmic expression of PDGF-B/PDGFR-β and VEGF/VEGFR2 was detected in fusion cells. [file 12885_2019_6460_MOESM5_ESM.tif]

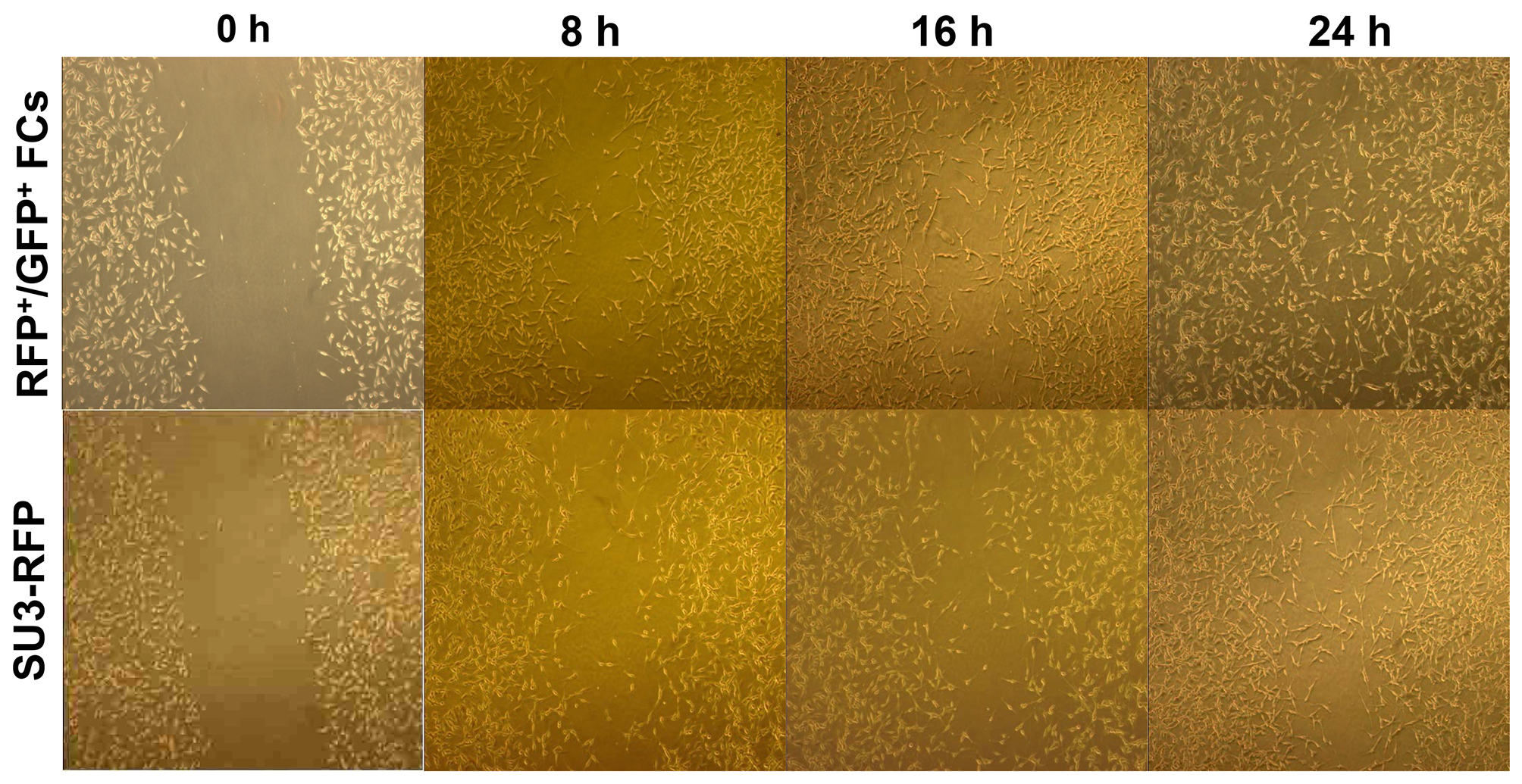

Supplement: Supplementary file 6 — Additional file 6: Figure S6. Cell monolayers were wounded by scraping with a sterile pipette tip, then washed twice to remove detached cells and debris, and the size of wound was observed every 6 h. The results revealed that fusion cells possessed increased migration ability. [file 12885_2019_6460_MOESM6_ESM.tif]
